# Relation among Perceived Weight Change, Sedentary Activities and Sleep Quality during COVID-19 Lockdown: A Study in an Academic Community in Northern Italy

**DOI:** 10.3390/ijerph18062943

**Published:** 2021-03-13

**Authors:** Margherita Micheletti Cremasco, Anna Mulasso, Alessia Moroni, Andrea Testa, Raffaella Degan, Alberto Rainoldi, Emanuela Rabaglietti

**Affiliations:** 1 Department of Life Science and Systems Biology, University of Torino, Via Accademia Albertina, 13, 10123 Torino, Italy; margherita.micheletti@unito.it (M.M.C.); alessia.moroni@unito.it (A.M.); 2 NeuroMuscular Function Research Group, Department of Medical Sciences, University of Torino, P.zza Bernini, 12, 10143 Torino, Italy; alberto.rainoldi@unito.it; 3 School of Exercise and Sport Science, University of Torino, P.zza Bernini, 12, 10143 Torino, Italy; andrea.testa886@edu.unito.it; 4 Suism University Service Center in Hygiene and Sport Sciences, University of Torino, P.zza Bernini, 12, 10143 Torino, Italy; raffaella.degan@unito.it; 5 Department of Psychology, University of Torino, Via Verdi, 10, 10124 Torino, Italy; emanuela.rabaglietti@unito.it

**Keywords:** weight changes, sedentary activities, sleep quality, COVID-19, workers, students, lifestyle, academic community, adult population, young adult population

## Abstract

In Italy, COVID-19 lockdown was imposed from 8 March until 3 May 2020 with negative consequences on the lifestyles and health of people. Within this context, the paper aims: (i) to analyse the impact of COVID-19 lockdown on perceived weight changes; (ii) to evaluate factors associated with the perception of weight changes (Body Mass Index (BMI), sleep quality, time spent in sedentary activities), in an Italian academic community of students and workers. A total of 3666 participants took part in this cross-sectional study (2838 students and 828 workers, of whom 73.0% were female). *T*-test, Chi-square test and the two-way ANOVA were used. Results showed that 43.3% of participants perceived a weight gain. Workers experienced a more substantial increase in body weight (0.7 kg) compared to students (0.3 kg; *p* = 0.013). A significant difference between preobese/obese workers (0.9 kg) and students (−0.3 kg; *p* < 0.001) was found. Overall, 57.0% of the sample was characterized by high levels of sedentary activities. Sedentary people noticed a higher weight gain (0.4 kg) compared to less sedentary people (0.3 kg; *p* = 0.048). More than 45% of participants reported a worsening of sleep quality and showed a perceived increase in body weight (0.5 kg) in comparison to those who improved their sleep quality (no weight change; *p* = 0.001). Designing tailored interventions to promote health-related behaviours during lockdown periods is essential.

## 1. Introduction

On 11 March 2020, the WHO defined the Severe Acute Respiratory Syndrome-Coronavirus-2 (SARS-CoV-2) infection as a pandemic [1]; a few days earlier Italy, the first heavily affected nation in Europe after COVID-19 spread from China [2], had already adopted extraordinary measures of social containment by legally imposing a national lockdown through a decree [3]. Prolonged mandatory social distancing was chosen according to scientific evidence that the main route of transmission of the virus was via direct and indirect contact through air, saliva and respiratory secretions, droplets and aerosol, emitted by infected subjects [4], as pointed out in March WHO recommendation and confirmed [5,6].

Lockdown actions, which inhibited people’s movements and social activities, were considered suitable right from the beginning in Italy [7], in Spain a few days later [8] and other countries such as China and South Korea also adopted them in the early stages of the spread of this disease in order to limit viral transmission and to prevent, or minimize, the impact of infectious disease, to avoid the collapse of health facilities and to contain deaths [9].

To contain the virus diffusion, the preventive measures adopted to reduce human interactions were illustrated by Italian government: individuals were required to not leave their houses, not just as a “quarantine” for those who suspected being infected, but there was effectively an order for every single person to “stay at home” (translated as the #stayathome decree) [3,10]. In Northern and Central Italy during COVID-19, the lockdown condition was imposed from 8 March 2020 [11] and was immediately extended to the entire national territory [3] and lasted until 3 May 2020 [12]. Forbidding both long-distance travels and short trips, closing shops, restaurants, schools, workplaces and any service considered nonessential, social distancing, and isolation at home suddenly reduced the amount of daily activities one could do, altered lifestyle and dietary habits, and additionally caused social and economic consequences. Different studies have pointed out how extended lockdown periods can adversely affect human health and wellbeing [9,13] with an increasingly sedentary lifestyle having negative consequences on psychophysical conditions and life quality [14]. Physical inactivity is an important research topic in social isolation conditions, as it is associated with a higher risk of cardiovascular and metabolic disease onsets that may predispose one to a greater risk of severe illness from COVID-19 [15,16,17]. A series of restrictive measures were imposed with the closure of gyms and sport centres and the ban of the majority of outdoor and social activities, with restrictions on walking distance. Therefore, a drastic reduction in physical activity occurred without adjusting dietary habits to lockdown conditions, resulting in weight gain and unhealthy consequences [18].

Containment measures against COVID-19 increased sedentary behaviours, modifying use of time, diet and also sleeping habits [19]. A recent review by Stockwell and colleagues [20] documented a worldwide trend concerning the increase in sedentary behaviour during the pandemic lockdown, ascribed to both work conducted from home and activities in front of a screen, such as watching television, playing video games and reading during free time. Several recent studies investigated how lifestyle habits changed in Italy during the national lockdown period—for example, Cancello and colleagues distributed a self-report survey in Northern Italy and pointed out that 68% of the sample experienced reduced physical activity, 42% increased their food intake and 43% showed symptoms of insomnia [21]. Increasing the amount of time spent in sedentary activities is also associated with the daily conditions of digital-education, e-learning and smart working [19]. In particular, a study of Cellini and colleagues [22] reported that, starting from the second week of lockdown, young adults (both students and workers aged 18–35) stated they had changed their time management, increasing the use of digital media and spending more time in bed, and reported a poorer sleep quality. Regarding the latter topic, Siversten and colleagues [23] documented a high and growing prevalence of sleep problems and insomnia in young adults; it is thus known that an extensive use of media devices interacts with sleep, especially in the two hours before bedtime [24]. In quarantine and isolation periods, nutritional habits changed because of the limited access to food, caused by the restrictions in grocery shopping. This reduced availability consequently led to a diet switch to unhealthy food, poor in fresh fruit and vegetables [25]. When individuals respond to stress by eating more, the selected foods are typically high in sugar and fat, and people also drink alcohol to feel better, leading to weight gain and negative health consequences [26].

Food choices and meal pattern changes were documented as more unhealthy during the COVID-19 confinement periods in several parts of the world, with widespread evidence of an upsurge in the intake of unhealthy food and out of control eating, associated with a documented decline in physical activity and increased sedentary (sitting) behaviours [27]. Zupo and colleagues [28] identified a common trend about dietary behaviours during the COVID-19 lockdown in many countries; while data concerning consumption of meat and junk food are not coherent, a moderate increment in the consumption of fruit, vegetables and pulses emerged in many cases, but a general decline in the overall dietary lifestyle spread significantly. A higher consumption of snacks and carbohydrate sources, especially those with a high glycaemic index (such as homemade bread, pizza and desserts) was also recorded, and as the authors underlined, an unhealthy dietary habit facilitates poor control of body weight. It is commonly accepted that quarantine led to a worsening of the quality of diet and an increase in the intake of almost all food categories with potential future negative effects on health [29].

It is known that obesity is related not only to abnormal eating behaviours, but also to stress and sleep deprivation [30]. Lockdown conditions can lead not only to alterations in food choice, timing, and quantity, but also loss of sleep quality. The disruptions to sleep quality and quantity, together with physical inactivity, induce decreased rates of skeletal muscle protein synthesis and impair insulin resistance, compromising immune defence [31]. Significant changes in sleep quality, quantity and timing associated with COVID-19 home confinement were demonstrated, and consequently changes in lifestyle habits occurred [32]. A survey conducted during the last 14 days of the Italian lockdown has already highlighted effects on sleep quality with disturbances such as insomnia associated with symptoms of depression and anxiety [33].

Muscogiuri and colleagues [34] have recently pointed out that quarantine-related stress determines sleep disturbance and increases in food consumption. They pointed out that the desire to consume a specific kind of food (for example, carbohydrates, with a high glycaemic index), encourages the production of serotonin and determine a positive effect on mood. 

In Italy, after about a month of lockdown, a greater consumption of sweets, bread, pizza undeniably occurred, with consistent weight gain and minor adherence to the Mediterranean diet, mostly by people over 30 [19]. This occurred because, compared to an increased intake and generally unhealthy food choices changes, a decrease in physical activities was often added. Decreasing physical activity level is recognized as one of the most serious problems induced by home isolation; worldwide, before the COVID-19 pandemic, insufficient physical inactivity was already described as a global public health problem [35]. In this regard, the World Health Organization [36] indicated physical activity as mandatory for the prevention of noncommunicable diseases, making it a long-term global program (2018–2030). In particular, despite the life conditions imposed by the 2020 pandemic, the WHO itself recommended to stay active and disclosed specific guidelines to maintain a healthy lifestyle during isolation and quarantine time, to preserve both mental and physical health [37]. Moreover, since the reduced physical activity, lack of dietary restraint and inadequate sleep are all recognised as risk factors for weight gain during quarantine [31,38], it is important to monitor weight as well as behavioural changes and tendency to be physically inactive due to the lockdown period.

Since it has been shown that physical activity, nutrition and sleep play fundamental roles in human health and physiology and that poor sleep, physical inactivity, and time spent indoors may be determined by a condition of isolation [31], we intended to evaluate the relationship between some of these aspects in the lockdown situation and weight conditions in a sample of students and workers of an academic community in Northern Italy. In particular, the first aim of the current research was to analyse the impact of home confinement during the COVID-19 lockdown on weight changes perceived over two months after the beginning of the isolation period. This aspect has been studied in general among the whole academic community, but the present study distinguishes between students and workers, hypothesizing the presence of differences in the two categories. Another aim was to evaluate factors associated with weight changes perception, such as the role of the Body Mass Index (BMI), the time spent in sedentary activities (time spent each day reading newspapers/watching news, watching movies and series, making video calls and browsing social networks) and sleep quality, analysing the differences between students and workers.

## 2. Materials and Methods

### 2.1. Procedure

A large cross-sectional online survey was distributed in May 2020 after two months of lockdown in Italy. Approval from the University of Torino’s Ethics Committee (Protocol number 179496) was obtained. 

A survey questionnaire was realized on the platform Google Modules and disseminated through the University of Torino’s (UniTO) internal communication channels. The the project was sent to the personal email of each student (more than 80,000) and workers (about 4000 among teaching and technical-administrative staff) of UniTO, with an invitation to participate anonymously through a direct link to the questionnaire. At the beginning of the online survey, informed consent information was presented to the subjects and they agreed to take part in the study. Involvement was voluntary and without compensation. No exclusion criteria were established.

The whole sample was in the same situation of statutory confinement at home for at least two months, as the legal prescription was applied to every person at the national level. The questionnaire remained open from 14 May to 31 May 2020.

### 2.2. Measures

The survey covered the following areas:

- socio-demographic characteristics such as age, gender, educational level, occupation;
- self-declared anthropometric data such as height and weight at the time the questionnaire was compiled (from 14 May until 21 May 2020), and weight prior the lockdown in order to evaluate the weight changes perceived after more than two months of confinement (since the lockdown lasted from 9 March to 4 May 2020 and the questionnaire was closed 31 May). Weight before the lockdown was useful in calculating Body Mass Index (BMI) to evaluate the incidence of weight classes using BMI international cut-off values [39], and specifically: <18.5 kg/m^2^ underweight, 18.5–24.9 kg/m^2^ normal weight, and ≥25 kg/m^2^ overweight/obese;
- information on lifestyle habits concerning the amount of time spent in sedentary activities in a week (answer options were: Never, 1 h, 2/3 h, 4/5 h, 6/8 h and >8 h), referring to SIT-Q by Wijndaele and colleagues [40] detailing various potential static activities (with or without the use of digital media devices), such as reading newspapers/watching news; watching movies and series; making video calls; surfing social networks;
- sleep quality, with an ad hoc question to evaluate if the perception of sleep quality had changed (No; Yes, positive changes/negative changes) referred to the home confinement obligatory period, as was similarly carried out by other studies in the same context [21].

### 2.3. Statistical Analysis

Statistical analyses were conducted with the Statistical Package for Social Sciences (SPSS), version 26.0 (SPSS Inc., Chicago, IL, USA). Height and weight were detected and treated with an accuracy of 0.01 m and 0.1 kg, respectively. Level of significance was set at 0.05 for all tests. Descriptive statistics were performed for all the variables. For the analysis, answers related to each sedentary activity were grouped into three categories based on the duration of said activity: never/1 h, 2/5 h and >6 h. To identify any differences between students and workers, a *t*-test and chi-square test were used.

Firstly, to analyse whether gender and student/worker status interact with each other, and whether they have an effect on the perception of body weight change, a two-way ANCOVA was run. The same model was tested using BMI categories instead of gender.

Secondly, to identify groups who spent similar amounts of time during COVID-19 lockdown involved in some sedentary activity such as reading newspapers/watching news, watching movies and series, making video calls and surfing the social networks, a cluster analysis was carried out. The steps executed are as follows: hierarchical cluster procedure employing Ward’s method (squared Euclidian distance) and k-means clustering. Differences across cluster centres were presented using one-way ANOVA.

Lastly, two other models based on two-way ANCOVA were run. The first contemplated the existence of an interaction between clusters of sedentary activities and student/worker status, and the effect on weight change perception. The second introduced the sleep quality variable instead of clusters of sedentary activities.

## 3. Results

### 3.1. Characteristics of Participants

Table 1 shows baseline characteristics of the sample and compares students and workers. A total of 3666 people (mean age 29 years, SD = 12) took part in this study; 73% of the participants were women, a slightly higher percentage than the 61% representing the totality of women in the community of UniTo. The majority of the sample was represented by students (*n* = 2838; 77%), although the percentage of students enrolled in the University of Torino is even higher (more than 90%); 68.8% of the students of our sample were working towards a bachelor’s degree, marginally different from the 60% represented in the entire student population. Regarding workers, our sample was composed of 50.2% teachers and 49.8% technical-administrative staff—the latter amounts to a bit less than 50% of the UniTo community. Students and workers differed by age (24 years for students and 47 for workers). Regarding the level of education, 50.4% of the workers had postgraduate experience.

### 3.2. Weight Changes Perception

Results highlighted a higher overweight/obesity condition among workers (26.0% compared to 14.2% of students), while among students there is a higher underweight frequency (more than 11.0%) than workers (who do not arrive at 5.0%). More than two-thirds of participants perceived weight changes after about two months of home isolation (43.3% perceived a weight increase, 26.6% a weight reduction). On average, people reported an increase in body weight of 0.4 kg (SD = 2.3 kg), with significant differences (*p* = 0.013) between students (+0.3 kg) and workers (+0.7 kg). About half of the workers declared a perceived weight gain, while among the students there is the highest frequency of those who perceived a weight decrease (about 30.0%) (Table 1).

### 3.3. Sedentary Daily Activities

With respect to sedentary daily activities performed during COVID-19 lockdown, most of the participants reported they spent on average 2–5 h a week reading newspapers/watching news (50.7% of the whole sample), watching movies and series (46.8%), making video calls (44.7%), and surfing social networks (43.0%). Notable differences between workers and students were found regarding the practice of sedentary activities: differences were significantly higher among workers (29.2%) who spent more than 6 h a week reading newspapers/watching news compared to a higher frequency of students who devoted 6 h a week to watching movies and series, making video calls, but, most of all, surfing social networks (35.6% of students compared to 14.7% of workers). A significant number of participants (45.3%) reported a worsening of sleep quality. All the variables analysed resulted in statistical differences between students and workers (see Table 1).

### 3.4. Effects of Individual Characteristics on Body Weight Change Perception

The two-way ANCOVA showed a significant interaction of gender and student/worker status on weight change perception— F(1, 3662) = 9.72, *p* = 0.002 (Table 2). Specifically, simple main effects analysis showed that female students were different from female workers in terms of weight change perception (*p* < 0.001; female students and workers perceived an increase in body weight of 0.2 and 0.9 kg, respectively). No differences were detected between male students and workers (*p* > 0.05). Furthermore, female students presented a minor weight gain perception compared to male students (*p* = 0.020; 0.2 versus 0.4 kg, respectively), as well as female workers compared to male workers (*p* = 0.024; 0.9 versus 0.5 kg, respectively). A main effect of student/worker status on weight change perception was also found—F(1, 3662) = 10.21, and *p* = 0.001. There is no main effect of gender.

The same statistical analysis was repeated to investigate if there was an interaction of BMI category and student/worker status on the perception of weight change (Table 2). A significant interaction was indeed found—F(2, 3660) = 9.39, *p* < 0.001. Simple main effects analysis revealed that underweight (*p* < 0.001) and normal weight students (*p* < 0.001) were different in terms of weight change perception compared to obese students. Specifically, underweight and normal weight students perceived an increase in weight of 0.6 and 0.3 kg, respectively; preobese/obese students, on the other hand, perceived a reduction in weight of 0.3 kg. No differences were found among BMI categories in the subgroup of workers. Furthermore, normal weight (*p* = 0.001) and preobese/obese students (*p* < 0.001) were different from workers of the same BMI category. Normal weight students and workers perceived increases in their weights of 0.3 and 0.7 kg, respectively. The opposite happened in preobese/obese students (reduction of 0.3 kg) and workers (increase of 0.9 kg). A main effect of student/worker status was found—F(1, 3660) = 6.90, *p* = 0.009. No main effect of BMI on weight change perception was observed.

### 3.5. Effects of Sedentary Daily Activities during COVID-19 Lockdown and Sleep Quality on Body Weight Change Perception

A cluster analysis was carried out to identify people who spent similar amounts of time during COVID-19 lockdown in sedentary activities such as reading newspapers/watching news, watching movies and series, making video calls and surfing social networks. Using Ward’s method of hierarchical cluster analysis two clusters were detected. The cluster centres differed from each to other (*p* < 0.001). The first cluster is composed of 2088 people (57.0%) characterized by high levels of sedentary activities; while the second cluster consists of 1578 persons (43.0%), who spent little time in sedentary activities. The two resulting clusters were identified as “high” and “low” level of sedentary activities.

A two-way ANCOVA was run to investigate whether sedentary activities (two clusters) and student/worker status interacted among each other and whether they affected weight change perception (Table 3, first rows). The same analysis was then repeated regarding sleep quality instead of sedentary activities (Table 3, last rows). Results showed a major effect of the sedentary activities—F(1, 3662) = 3.91, *p* = 0.048, on weight change perception. Sedentary people (cluster 1) mentioned a more notable increase in weight (0.4 kg) compared to less sedentary people (cluster 2; 0.3 kg). Likewise, the main effect of student/worker status was found—F(1, 3662) = 26.19, *p* < 0.001. No interaction effects were found. Similarly, changes in sleep quality had a major effect on weight change perception—F(2, 3660) = 6.32, *p* = 0.002. People whose sleep quality had worsened described a perceived weight gain (0.5 kg), as opposed to those who had instead improved their sleep quality (in this subgroup no change of weight was observed). Also the main effect of student/worker status was present—F(1, 3660) = 21.80, *p* < 0.001. Interaction effects were not significant.

## 4. Discussion

Our study, carried out through an online questionnaire which was filled out after more than two months of home confinement during the COVID-19 lockdown, highlighted the effects on weight, mostly increases, in an academic community in Northern Italy, distinguishing between students and workers. We investigated the relationship of substantial changes affected by the status of student/worker, in the same sociocultural context, with the time spent in sedentary activities/behaviour and changes in sleep quality. The fact that more than 70% of the answers were expressed by a female sample must be taken into account. A higher female component in the sample may be related not only to a higher percentage in the UniTo community, but also to a different response to the investigation by the two genders. This peculiarity had already emerged in other similar surveys conducted in the context of problems resulting from the COVID-19 pandemic in Italy and other countries, both among students [41,42], and in general in adult population samples (about 73–77% females in several studies [19,22,43,44]). From analysing the anthropometric declared data, the perception of weight gain was true for 43.3% of the sample, in accordance with results found in another Italian national survey (48.6%) [19]. Scarmozzino and Visioli [45] claimed that only 19.5% of their sample gained weight and closed the questionnaire by 15 April, after only one month of lockdown. 

The perceived weight gain is more consistent and frequent among workers compared to students. With a distinction by gender, female workers perceived the highest body weight gain, though female students noted the lowest increase, while male students and workers experienced about the same increase (less than 0.5 kg).

Overweight/obesity conditions were roughly doubled among workers (26.0%) compared to students, while the contrary occurred regarding underweight frequency (11.3% in students). The tendency of those who are already overweight/obese to gain even more weight during lockdown is supported by other Italian surveys. Marchitelli and colleagues [46] conducted a study on people affected by overweight/obesity: the percentage of the sample that reported weight gain during the COVID-19 lockdown was higher than 50% in overweight people and 66% among obese subjects. Pellegrini and colleagues [43] pointed out a mean average of 1.5 kg increase on an exclusively obese sample. Barrea and colleagues [47] in a sample of adults from Southern Italy, about 90% of whom were already overweight/obese before lockdown, showed a mean of 1.8 kg weight gain after 40 days of quarantine.

Analysing our results, we highlighted a different tendency depending on worker/student status because our sample’s preobese/obese workers increased their weight of 0.9 kg, while preobese/obese students declared a reduction of 0.3 kg. It should also be highlighted that the problem of excess and weight gain is greater than it appears, as it is known that the use of self-reported weight and height usually leads to an overestimation of height and underestimation of weight, and consequently BMI and the prevalence of overweight and obesity are underestimated as well [48]. Even if a general weight gain occurred during first 2020 lockdown in Italy, the present research pointed out that 26.6% of the sample reported a loss of weight. This percentage is higher than the 13.9% found by Di Renzo and colleagues [19], but the difference could be due to the fact that their questionnaire covered a shorter time from the first phase of lockdown (survey open from 5 April until 24 April). On the contrary, our questionnaire was filled in in more than two months since the lockdown inception; we can explain this higher percentage as the weight loss process being slower than a weight gain process. We can also underline that Di Renzo and colleagues’ sample [19] included subjects from all over Italy (about one-third of their sample was from Southern Italy, where a greater tendency of overweight and obesity is known). In our study about differences by student/worker status, the highest frequency of those who perceived a weight decrease is among the students (about 30%).

The present study highlighted an effect of sedentary activities and sleep quality on weight change perception. People who reported sedentary habits and worsening sleep quality perceived a more significant increase in weight. Sedentary activities had a major effect on weight change perception, with a more notable increase in weight compared to less sedentary people.

Regarding sedentary daily activities, time spent reading newspapers/watching news was higher among workers than students, who, on the contrary, surpassed workers in all others static activities, such as watching movies and series, making video calls, but most of all surfing social networks. Stockweel and colleagues [20] pointed out an increase in sedentary behaviours particularly regarding screen activity, not only in a university context, but also in different samples and countries. A rising trend of prolonged screen time may potentially have implications on physical and mental wellbeing [49].

In this regard, physical inactivity is one of the most important factors linked to the risk of weight gain, up to becoming overweight or developing obesity. Weight gain may be magnified by the unhealthy dietary habits that very frequently accompany prolonged television viewing [50].

We also add that a worsening of sleep quality was reported by less than the half of the whole sample, and among them there was a perceived increase in weight in comparison to those who improved their sleep quality (no weight change). The significant relationship between fewer hours of sleep and reported weight gain finds support in other studies on the effects of isolation for COVID-19 [38]. Regarding sleep disturbance, a survey conducted during the last 14 days of the Italian lockdown (between 19 April and 3 May 2020) through an online questionnaire, distributed to the general population the same way as in the present study, found that 42.2% (in a sample of about 1500 subjects) suffered from sleep disturbances and, among them, 17.4% reported moderate/severe insomnia [33]. In another survey conducted among the general adult population, in Northern Italy from 15 April to 4 May 2020 [21], 43% of the whole sample declared a worsened sleep quality perception, a slightly lower frequency than the present study. Our results are higher and concern a longer period (after two months of isolation) with respect to Gualano and colleagues [33] and Cancello and colleagues [21]. We found a higher frequency of sleep quality decline in students (46.7% as opposed to 40.2% among workers). Poor sleep quality is frequently mentioned by students [51]; this issue can increase during lockdown periods and it is more serious for those with anxiety, depression and stress disorders which tend to intensify in isolation [29,52,53]. In this regard, according to Cellini and colleagues [22], among Italian young adults during isolation there was an increase in the quantity of sleep but a worsening of the quality that was stronger especially among those suffering from anxiety, depression and stress (though the study by Cellini and colleagues concerned only the second week of lockdown and the sample included young people aged 18–35, students and workers together). In addition, in another Italian study, Marelli and colleagues [54], using a sample of university students and administrative staff, pointed out that the impact of COVID-19 lockdown on sleep quality was greater in students (insomnia from 6.9% pre-COVID to 16.3% during COVID) than in administrative workers, as well as the anxiety and depressive symptoms. Similarly, after two weeks of confinement in France, about one-third (more than 100 subjects) of the sample reported trouble sleeping, with greater frequency or severity among young adults (18–35 years) [55]. Hartley and colleagues [56] on a sample of 1777 French adults (of which 72% was aged 25–54) underlined a poor sleep quality. In a study conducted in 49 countries, researchers found that 40% of the participants reported a decreased sleep quality in contrast with the time before COVID-19 pandemic, underlining the need for proper global interventions on sleeping aids [57]. 

In addition to depressive disorders, some authors (among others, Léger and colleagues [58]) also identified Video Display Terminal (VDT) use as a cause of sleeping problems: the bright blue light of screens has a well-known negative influence on sleep quality, not to mention that a continuous exposure to stressful information may contribute to increase anxiety and emotional distress. Additionally, it should be highlighted that the intensive usage of digital media near bedtime is known to contribute to poorer or disrupted sleep [24], particularly in young adults [23,59,60]. In our study, in fact, the students appeared to be more sensitive to the risk of sleep disturbance. Regarding workers, a contribution to the deterioration of sleep quality may be associated with smart-working, consistently with Barrea and colleagues [47]. In this case, blue light exposure due to extended screen time working remotely from home (as well as browsing the internet to keep up to date with news and COVID trends, or simply passing free time) may contribute to increased sleep disorders [58]. 

In accordance with the studies mentioned above, our research analysed the impact of COVID-19 lockdown on perceived weight changes in relation to the abovementioned factors, but for the first time comparing consistent numbers from both categories, students and workers in an academic community in Italy. Several studies explored the topics of interest on students or on workers alone, or on the general population and not defining composition. We have considered two different groups (students and workers) in the same community, which is supposedly the same socio-cultural environment, to compare lifestyle changes and criticisms to point out analogies and differences and to better address future policies and interventions in order to promote health and support wellness in our university community and in others.

Our study has some limitations. Among these, one of the most notable is the use of a self-assessment questionnaire, with some questions created ad hoc for specific situations and conditions: the anthropometric measures are declared, with the risk of misreporting data. Nevertheless, the risk is simply underestimatation, and consequently we believe the results obtained are substantial similarly to other web questionnaires that have been employed during COVID-19 isolation. It is noteworthy to underline that the perception of weight change can be influenced by many other factors (e.g., level of physical activity, dietary habits information, alcohol consumption, psychological health, etc…) that have not been analysed or verified in this study. Furthermore, the number of participants is different among subgroups (e.g., students/workers, women/men, BMI categories, etc…), with some subgroups (e.g., underweight workers) composed of a limited number of people. In some cases, differences in weight changes among subgroups are limited, although statistical significance is maintained. Lastly, the cross-sectional design of the study did not allow to analyse the long-term consequences of the lockdown.

This study has also some strengths. One above all is represented by the fact that the survey was conducted after two months of lockdown, and not just a few weeks after closing or during isolation, as it was previously shown/demonstrated that a prolonged confinement is considerably associated with a greater perception of weight change [61].

This is a snapshot illustrating a specific moment of the first national lockdown experienced in Italy, without being able to properly catch the changes over time in the variables considered with respect to the pandemic continue, even though it could be useful for future comparisons.

## 5. Conclusions

If home confinement during COVID-19 lockdown seems to be an efficient strategy to contain the increment of Sars-CoV-2 infection, at the same time it unequivocally changes daily habits. Likewise, if this condition continues for months, it leads to effects on the person in terms of health consequences such as weight gain, worsening night sleep quality and daily excess of sedentary activities.

Our research may be useful in developing person-tailored interventions to maintain health-related behaviours during lockdown periods, taking into account the different ways people respond to home confinement, to avoid adopting unhealthy lifestyles. In the academic community, during lockdown all the participants, both students and workers, needed to increase the amount of time spent using a VDT for remote lectures, smart-working and other activities related to this unique situation; however, they also increased sedentary activities in their free time, mostly via digital tools, albeit with different purposes and interests, but nevertheless with a series of consequences on the quality of sleep and weight gain.

The request for person-tailored interventions reflecting the different characteristics and needs of the academic community (workers and students) could provide excellent customisation tools in technology use and digital applications. Internet and smartphone applications may provide the initial choice menu based on specific situations, and their use may be recommended to: control diet and maintain personal ideal weight [25]; promote in-house exercise tutorials, customizable by age and specific conditions, and suggest specific activities/exercises to compensate the excess of time spent in a static posture (as in screen use for work or study purposes); decrease sedentary behaviours during VDT use (e.g., alternating sitting and standing while at a desk and taking regular breaks from sitting) [20]; if the lockdown rules allow it, suggest physical activity (online tools or organized events) in public spaces [62], promoting walking, running and cycling outdoors [20]; monitor sleep quality [63] and observe the amount of sleep suitable to each condition, suggesting avoiding the use of digital media devices in the hour before sleep [24]; reduce the use of light-emitting screens before bedtime [59]. 

Paying more attention to the use of one’s time while at home, limiting VDT activities but staying active and adequately practicing daily physical activities are essential behaviours to maintain mental and physical health, to contain weight gain and to maintain a good sleep quality. Starting from the emerged criticalities, with differences based on the conditions of our academic community during the lockdown for the COVID-19 pandemic, it will be possible to design customizable measures of improvement and study differentiated and individualized ad hoc paths/applications that can also be extended to other comparable communities and work contexts.

## Figures and Tables

**Table 1:** Individual characteristics and sedentary daily activities during COVID-19 lockdown for the whole sample, students and workers.

Variable	Whole Sample *n* = 3666	Students *n* = 2838	Workers *n* = 828	*p* Value ^†^
Age, years, mean (SD)	29 (12)	24 (6)	47 (11)	<0.001 ^†^
Gender, *n* (%) Female Male	2675 (73.0) 991 (27.0)	2097 (73.9) 741 (26.1)	578 (69.8) 250 (30.2)	0.020 ^§^
Level of education, *n* (%) Middle school, 8 years High school diploma, 13 years University bachelor’s degree, 16 years University master’s degree, 18 years postgraduate education (Phd, master’s, etc…), +18 years	8 (0.2) 2069 (56.4) 808 (22.0) 346 (9.4) 435 (11.9)	0 (0.0) 1953 (68.8) 769 (27.1) 98 (3.5) 18 (0.6)	8 (1.0) 116 (14.0) 39 (4.7) 248 (30.0) 417 (50.4)	<0.001 ^§^
BMI, kg/m^2^, mean (SD)	22.3 (3.8)	21.9 (3.4)	23.4 (4.5)	<0.001 ^†^
BMI, categories, *n* (%) Underweight Normal weight Preobesity/Obesity	358 (9.8) 2689 (73.3) 619 (16.9)	321 (11.3) 2113 (74.5) 404 (14.2)	37 (4.5) 576 (69.6) 215 (26.0)	<0.001 ^§^
Body weight change perception, kg, mean (SD)	0.4 (2.3)	0.3 (2.4)	0.7 (2.1)	0.013 ^†^
Body weight change perception, *n* (%) Increase No changes Decrease	1586 (43.3) 1104 (30.1) 976 (26.6)	1173 (41.4) 838 (29.5) 827 (29.1)	413 (49.9) 266 (32.1) 149 (18.0)	<0.001 ^§^
Time spent reading newspapers/watching news (hours/week), *n* (%) Never/1 h 2–5 h >6 h	1144 (31.2) 1857 (50.7) 665 (18.1)	968 (34.1) 1447 (51.0) 423 (14.9)	176 (21.3) 410 (49.5) 242 (29.2)	<0.001 ^§^
Time spent watching movies and series (hours/week), *n* (%) Never/1 h 2–5 h >6 h	596 (16.3) 1717 (46.8) 1353 (36.9)	427 (15.0) 1329 (46.8) 1082 (38.1)	169 (20.4) 388 (46.9) 271 (32.7)	<0.001 ^§^
Time spent making video calls (hours/week), *n* (%) Never/1 h 2–5 h >6 h	1463 (39.9) 1638 (44.7) 565 (15.4)	1074 (37.8) 1309 (46.1) 455 (16.0)	389 (47.0) 329 (39.7) 110 (13.3)	<0.001 ^§^
Time spent surfing the social networks (hours/week), *n* (%) Never/1 h 2–5 h >6 h	959 (26.2) 1576 (43.0) 1131 (30.9)	576 (20.3) 1253 (44.2) 1009 (35.6)	383 (46.3) 323 (39.0) 122 (14.7)	<0.001 ^§^
Changes in sleep quality, *n* (%) No Yes, positive changes Yes, negative changes	1541 (42.0) 466 (12.7) 1659 (45.3)	1165 (41.1) 347 (12.2) 1326 (46.7)	376 (45.4) 119 (14.4) 333 (40.2)	0.004 ^§^

Notes: ^†^ based on independent *t*-test for continuous variables; ^§^ based on chi-squared test for categorical variables.

**Table 2:** Weight change perception divided by student/worker status and gender (first rows) and Body Mass Index (BMI) categories (last rows).

**Weight Change Perception**	**Students**			**Workers**			**Interaction** **F, *p*-Value**
	**Men** **(*n* = 741)**	**Women** **(*n* = 2097)**		**Men** **(*n* = 250)**	**Women** **(*n* = 578)**		
mean ± SD	0.4 ± 2.5	0.2 ± 2.3 *^,‡^		0.5 ± 2.6	0.9 ± 1.9 ^‡^		9.72; 0.002
**Weight Change Perception**	**Underweight** (***n* = 321**)	**Normal Weight** (***n* = 2113**)	**Preobesity/Obesity** (***n* = 404**)	**Underweight** (***n* = 37**)	**Normal Weight** (***n* = 576**)	**Preobesity/Obesity** (***n* = 215**)	**Interaction** **F, *p*-Value**
mean ± SD	0.6 ± 1.8 *	0.3 ± 2.2 *	−0.3 ± 3.5	0.3 ± 1.0	0.7 ± 1.8 ^‡^	0.9 ± 3.0 ^‡^	9.39; <0.001

Notes: Weight change perception measured in kg; (second row) * *p* < 0.05 versus workers of the same gender; ^‡^ *p* < 0.05 versus male within the same category (students/workers); (third row) * *p* < 0.05 versus preobese/obese students; ^‡^ *p* < 0.05 versus students within the same BMI category.

**Table 3:** Effects of sedentary activities during COVID-19 lockdown and sleep quality on weight change perception.

**Sedentary Activities**	**Weight Change Perception** **Mean ± SD**	**F, *p*-Value**
High level (cluster 1)	0.4 ± 2.4	3.91; 0.048
Low level (cluster 2)	0.3 ± 2.3	
**Sleep Quality**	**Weight Change Perception** **Mean ± SD**	**F, *p*-Value**
No	0.4 ± 2.1	6.32; 0.002
Yes, positive changes	0.0 ± 2.4	
Yes, negative changes	0.5 ± 2.5 *	

Notes: Weight change perception measured in kg; * *p* < 0.05 versus Yes, positive changes.

## Data Availability

The data presented in this study are available on request from the corresponding author.
